# Antioxidant, Cytotoxic, and Antibacterial Activities of the Selected Tibetan Formulations Used in Gandaki Province, Nepal

**DOI:** 10.1155/2021/5563360

**Published:** 2021-07-27

**Authors:** Madan Dhakal, Prakash Poudel, Upma Jha, Suresh Jaiswal, Khem Raj Joshi

**Affiliations:** ^1^School of Health and Allied Sciences, Faculty of Health Sciences, Pokhara University, Pokhara 33700, Nepal; ^2^Department of Pharmacy, Novel Academy, Pokhara 33700, Purbanchal University, Nepal

## Abstract

**Materials and Methods:**

An open-ended and semistructured questionnaire was used for an ethnomedicinal survey of the Tibetan formulations practiced in four Tibetan refugee settlements in Gandaki Province, Nepal. Based on the ethnomedicinal survey data, commonly used nine formulations were selected (Aru-18, Basam, Dadue, Dashel, Mutik-25, Raab Ga Yangzin Tea, Serdok-11, Sugmel-10, and Yungwa-4) to test biological activities. Antioxidant activity was evaluated using the 2,2-diphenyl-1-picryl-hydrazyl (DPPH) radical scavenging method. The cytotoxicity was examined by using the *Allium cepa* L. root tip meristem model. Similarly, the antibacterial effect was assessed by using well diffusion and broth dilution methods.

**Results:**

An ethnomedicinal survey showed a total of 52 Tibetan formulations were generally used by respondents for common diseases such as stomach disorders, diabetes, and migraine. From the antioxidant activity test, Sugmel-10 showed the highest DPPH free-radical-scavenging activity (IC_50_ 1.8 *μ*g/ml) and Yungwa-4 showed the lowest activity (IC_50_ 5.2 *μ*g/ml). Also, from the cytotoxic activity, the *A. cepa* root meristem model exhibited significant dose- and time-dependent growth suppression in Basam, Dadue, Mutik-25, and Serdok-11 as compared with cyclophosphamide standard drug. Similarly, Basam showed a good antibacterial effect having MIC 20 mg/ml and MBC 100 mg/ml against *Enterococci faecalis. Conclusion*. Research showed that Tibetan people preferred Tibetan formulations for the treatment and mitigation of several diseases. The result of antioxidant, cytotoxic, and antibacterial activities experimentally justified the ethnomedicinal value of nine common formulations (Aru-18, Basam, Dadue, Dashel, Mutik-25, Raab Ga Yangzin Tea, Serdok-11, Sugmel-10, and Yungwa-4). To the best of our knowledge, this study was performed for the first time in Nepal. Results from this preliminary study open the door to the scientific world to perform extensive pharmacological studies for designing and developing new therapeutic agents.

## 1. Introduction

Tibetan medicine (TM) is one of the earliest-known traditional medicines, and its history goes back approximately 2,500 years. In this system of medicine, a Tibetan doctor formulates an anticipative diagnosis and personalized treatment plan, where the treatment may last several months to years for chronic diseases [1]. In TM, particular treatment is codified in the form of sacred texts or pharmacopeia elucidated with the Buddhist understanding of herbal remedies [2]. There are more than 20 different dosage forms in traditional TM such as pills, powders, decoctions, lotions, ointments, and medicinal liquors [3]. Dried raw materials are ground, mixed homogenously, and ultimately pressed into pills, powder, or decoctions. Mantras are chanted to enhance the potency of the remedy in the course of blending [4]. In a particular formula, ingredients are blended as a dry powder where galenical forms are chiefly pills and medicinal powders in comparison to medicinal butter, plasters, and decoctions. An entire formula can be considered as a pharmacologically active entity with distinct pleiotropic effects [5]. The dosage form of this system constitutes several ingredients, which is through combinations of up to 108 or more ingredients. The governing research concerning multi-ingredient formula came from the Padma, a Swiss pharmaceutical company that develops standardized herbal formulas that originated from Tibetan medical knowledge [6–9].

Along with TM, traditional Iranian medicine (Persian medicine), traditional Chinese medicine (TCM), and Ayurveda are very popular in Asian countries. All these traditional systems of medicine use herbal drugs or extracts, acupuncture, massage, diet therapy, physical activity, and exercise. Like TM, Persian medicine, TCM, and Ayurveda follow humoral theory. Historically, different formulations and potential herbs are used in the abovementioned traditional system of medicines as evidence-based therapy. The integration of their principles, techniques, medication, and knowledge with modern medical sciences is the field of tremendous ongoing efforts and interests to develop new therapeutic options in current medicine. The effective management as suggested by traditional medicines regarding the human body is based on maintaining balance in body fluids and temperament, along with therapeutic and supportive strategies [10–13].

Antioxidants antagonized the damaging effects of free radicals and helped to prevent or repair that deleterious phenomenon in living cells [14]. Bioactive phytochemicals present in traditional medicines possess antimutagenic, anticancer, and antioxidant properties that provide a protective effect against various kinds of cellular injury. For instance, phenolic compounds (caffeic acid and *ρ*-coumaric acid) and flavonoids (kaempferol) are responsible for antioxidant activity; terpenoides (*ρ*-cymene and *γ*-terpinene) and essential oil (cuminaldehyde) are liable for antimicrobial effects [15, 16]. Also, natural antioxidants present in various plants decrease oxidative damage and help in inhibiting aging, mutagenesis, and carcinogenesis considering their radical scavenging activities [17]. The *A. cepa* root tip meristem model has been extensively used for the assessment of antimitotic and cytotoxic properties [18–22] by utilizing the growing roots of *A. cepa*. The cell division in its meristematic cells resembles normal human cancer cell division. Thus, these meristematic cells can be used in the analysis of drugs with possible human anticancer activity [23]. Using plant extract to treat infections is an ancient practice in traditional medicine. For this intent, humans have used natural products derived from plants, animals, and microbial sources for millennium either in the crude extracts or pure forms [24]. Plant secondary metabolites are predominately liable for their antimicrobial activity [25]. Likewise, many animals and their products have been used in traditional medicines across numerous medicinal systems [26–28]. In recent years, indiscriminate uses of antibiotics have generated the problem of antibiotic resistance. Similarly, the quest for new antimicrobial agents is a worldwide concern as herbal medicine from natural sources showed lesser side effects than synthetic medicines. Furthermore, some traditional TM may suggest promise in clinical treatments as plants, animals, trace elements, and minerals are the abundant sources used in such traditional medicines including biologically active substances and amino acids. Plants used are mostly cold and drought resistant, and they perform thorough photosynthesis [3].

Tibetan traditional medicines are being practiced more in the Himalayan region and are recommended by Amchis (Tibetan medicinal practitioners) in the northern belt of Nepal, the border of Tibet (autonomous region of China). Tibetan people residing in the country and families in the Tibetan refugee camp more preferred these formulations. This system of medicine is not normally assessed by the people in the central or southern parts of the country. This may be due to the limited number of general health practitioners available in that region or due to the insufficient scientific evidence of these traditional medicines.

Antioxidant, cytotoxic, and antibacterial studies of nine common Tibetan formulations (described briefly in Table 1) have not yet been studied and justified. Therefore, in this study, we performed the ethnomedicinal survey, and based on the survey data, we collected nine formulations and evaluate their effectiveness against antioxidant, cytotoxic, and antibacterial activity.

## 2. Materials and Methods

This research was conducted in two stages. The ethnomedicinal survey was carried out in the first stage, and in the second stage, the study of the biological properties of commonly used formulations was conducted.

### 2.1. Study Area and Data Collection

The ethnomedicinal survey on Tibetan formulations was conducted in four Tibetan refugee settlements (Jampaling, Paljorling, TashiLing, and TashiPalkhel) from August 2016 to January 2017 in the Gandaki Province of Nepal. The study was performed by taking the ethical approval and following the ethical guidelines approved by the Pokhara University Research Center and the Institutional Review Committee, Pokhara University. Data on the TM were recorded by using a questionnaire (open ended and semistructured) form by face-to-face interviews with 80 respondents when they visit Amchis. Respondents were interviewed in the Nepali language after taking their verbal consent. Mostly, the interviews were usually initiated in the form of informal discussions to boost the confidence of the interviewees, and we review their prescriptions. All the data from 80 respondents were analyzed; then, we found that stomach disorders, diabetes, and migraine were common diseases with high frequencies. Then, we focused primarily on the data on the formulations used in these ailments. So, we collected three mostly used samples from each disease for the study of biological activities. Those samples were identified with the help of Amchis.

### 2.2. Chemicals and Reagents

2,2-Diphenyl-1-picryl-hydrazyl (DPPH) and ascorbic acid were from Wako Pure Chemical Industries, Ltd., Osaka, Japan, and Qualigens Fine Chemicals Pvt., Ltd., Mumbai, India. The chemicals used in the analysis were of analytical reagent grade, and all the glassware used were from Borosil Glassworks Ltd., Mumbai, India. Cyclophosphamide was obtained as a gift from Manipal College of Medical Sciences, Phulbari, Pokhara, Nepal. All bacterial media such as Mueller Hinton Agar (MHA) and nutrient broth were from HiMedia, Mumbai, India.

### 2.3. Antioxidant Activity

Formulations were collected from two clinics (Paljorling branch ward no. 9 and Tashi Ling branch ward no. 17) situated in the Pokhara Metropolitan city of Kaski District. Collected formulations were ground into a fine powder using a pulverizer. Each powdered drug was extracted twice with 100% ethanol for 24 hrs at room temperature. Then, it was filtered and concentrated to dryness using a rotary evaporator and a vacuum desiccator under reduced pressure, and thus, the obtained dry extracts were stored in the refrigerator at 2–8°C for further use.

100 *μ*m of DPPH solution was prepared by dissolving 39.432 mg of DPPH free radical in 1000 ml ethanol. Ethanolic extract of each sample was weighed and dissolved in ethanol to make the stock solution of 1 mg/ml. Serial dilution was performed with 99.9% ethanol to make test samples of each extract and ascorbic acid in the range 0.1 *μ*g/ml, 1 *μ*g/ml, 10 *μ*g/ml, and 100 *μ*g/ml concentration, respectively.

DPPH free-radical assay was performed according to the method given in [33] with some modifications. In brief, 4 ml of the sample solution of various concentrations (0.1 *μ*g/ml, 1 *μ*g/ml, 10 *μ*g/ml, and 100 *μ*g/ml) of the sample was mixed with 4 ml of the DPPH solution (100 *μ*m). The mixture was allowed to stand for 30 min in the dark for completion of the reaction. The absorbance was observed at 517 nm by using a UV spectrophotometer.

### 2.4. *In Vitro* Screening of Cytotoxic Activity

The *A. cepa* root tip meristem model was used to examine the cytotoxic activity [19, 34] with minor modifications. Different formulations were separately ground into a fine powder using a pulverizer. Each powdered sample was extracted with ethanol for 24 hrs at room temperature. The bulbs of onion (*A. cepa* 50 ± 20 g) were grown in the dark place over 100 ml distilled water at room temperature till the roots have grown to 2-3 cm length. Later, the bases of each of the bulbs were suspended on different concentrations of the sample extract within 100 ml beakers and incubation was carried out. Then, the best-developed root length of each onion in each group was measured and the mean root length was calculated. Also, the number of roots at 0, 24, 48, 72, and 96 hrs for each concentration of extract and control was determined. The percentage root growth inhibition after treating with the extract at 24, 48, 72, and 96 hrs was determined and compared with that of control bulbs. The positive control drug cyclophosphamide and four samples were taken at the concentration of 1 mg/ml and 10 mg/ml.

### 2.5. Antibacterial Activity

Four bacterial strains, i.e., two Gram-negative bacteria (*Klebsiella pneumoniae* and *Pseudomonas aeruginosa*) and two Gram-positive bacteria (*Staphylococcus aureus* and *Enterococcus faecalis*), were purchased and subcultured at nutrient agar slants at 4°C for routine use. Active cultures for experiments were prepared by transferring a loopful of cells from the stock cultures on the surface of the agar in Petri dishes and then incubated for 24 hrs at 37°C.

*In vitro* antimicrobial activity was screened by using a well-diffusion method explained in [35] with some modifications. Mueller Hinton Agar (MHA) medium was poured aseptically into sterilized Petri dishes (20 ml) to solidify and was allowed to dry for 15 mins. Wells (6 mm and about 20 cm apart) were made in each of the seeded agar in Petri dishes using sterile glass Pasteur pipettes and labeled properly. Stock solutions of each formulation were prepared at a concentration of 100 mg/ml in 10 ml distilled water. About 100 *μ*l of formulations extract were added by using a micropipette into the wells and allowed to spread at room temperature for 1 hr. The sterile cotton swab was used on the surface of the MHA plate. Cefpodoxime and ofloxacin were used as standard antibiotics. Media plates were then incubated at 37°C for 24 hrs in an incubator. The diameter of the inhibition zone was calculated on the next day. The extract having antibacterial potency inhibits the growth of bacteria around the well. The larger the zone of inhibition, the higher the antibacterial activity.

The MIC testing was performed for the extracts having the highest zone of inhibition (20 mm or greater in diameter) against at least one test organism. The MIC values were measured by the broth dilution method. The maximum dilution of extract that maintained an inhibitory effect resulting in no growth or absence of turbidity is known as MIC [36]. Selected extracts were subjected to dilution at different concentrations (150, 120, 100, 80, 50, and 20 mg/ml) using sterile nutrient broth medium as the diluent to prevent the growth of *S. aureus* and *E. faecalis*. Stock cultures of both microorganisms were separately prepared in 100 ml nutrient broth. 1.3 g of nutrient broth was completely dissolved in 100 ml distilled water and was sterilized in an autoclave. After cooling down, a loopful of bacterial cells were transferred from the bacterial subcultures plate to conical flasks containing 100 ml nutrient broth and incubated at 37°C for 24 hrs.

Similarly, the maximum dilution showing at least 99% inhibition is taken as MBC [37]. In MBC determination, dilutions and inoculations are prepared in the same manner as described for the determination of MIC.

### 2.6. Statistical Analysis

Data entry and analysis were carried out in Microsoft Excel Worksheet 2010. All experiments were performed in triplicates, and data were expressed as mean ± standard error of the mean. The linear regression equation was used to calculate the IC_50_ value. One-way ANOVA using Dunnett's multiple comparison test was employed for statistical analysis in GraphPad 5 for cytotoxic activity. Test values of *p* < 0.05, *p* < 0.01, and *p* < 0.001 were considered as significant, more significant, and highly significant, respectively [38].

## 3. Results

### 3.1. Ethnomedicinal Survey

**Figure 1:**
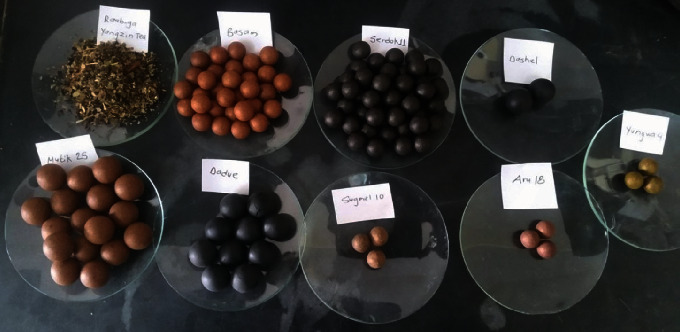
Selected nine different formulations for biological activity analysis.

From 80 respondents, we found that Amchis frequently prescribed fifty-two formulations (Agar-15, Agar-35, Ahuvi-25, Artse, Aru-10, Aru-24, Aru-18, Badkan tea, Basam, Chinni-Aru-18, Choe- 6, Chong-6, Dadue, Daemyuk, Dali-16, Dali-18, Dashel, Duetse-11, Dutik, Gonyod- 5, Goyu-18, Gur-13, Gur-18, Kyuru-25, Manu, Mappa, Men-1, Mengjor, Mutik-25, Nile, Noorpu, Nyeekheel, Pang-15, Payrak, Raab Ga Yangzin Tea, Sae-3, Sebree dana, Sebru-4, Sebru-23, Serdo-45, Serdok-11, Sethang, Shing-8, Shiser, Silchoe-15, Sugmel-10, Thang-25, Tikta-25, Tonsee-21, Tukun, Yukar, and Yungwa-4) for the treatment of stomach disorders, diabetes, and migraine in four Tibetan refugee settlements. Among them, it was found that Dashel (12.5%), Basam (8.75%), Raab Ga Yangzin Tea (7.5%), and Dadue (7.5%) were commonly used formulations for stomach disorders. Similarly, Aru-18 (11.25%), Sugmel-10 (8.75%), and Yungwa-4 (5%) were extensively used in diabetes. Also, Serdok-11 (10%), Aru-18 (8.75%), and Mutik-25 (6.25%) were the most preferred formulation for the treatment of migraine (Table 2). Powders, pills, and syrups were the major dosage forms of the formulations. As the largest numbers of respondents were found for nine different formulations, these formulations (Aru-18, Basam, Dadue, Dashel, Mutik-25, Raab Ga Yangzin Tea, Serdok-11, Sugmel-10, and Yungwa-4) were selected to test biological activities (Figure 1).

### 3.2. Antioxidant Activity

In this study, most of the extracts showed potent free-radical-scavenging activity with about 90% inhibition starting from 10 *μ*g/ml concentration. Among the sample, Sugmel-10 exhibited the highest inhibition (IC_50_ 1.8 *μ*g/ml) and Yungwa-4 showed the lowest activity (IC_50_ 5.2 *μ*g/ml), respectively which is comparable to ascorbic acid (IC_50_ 4.93 *μ*g/ml) (Table 3). Thus, data revealed that these formulations possess powerful antioxidant compounds that can be used for the treatment and prevention of oxidative stress-induced diseases.

### 3.3. Cytotoxic Activity

The cytotoxic effect of the ethanolic extract of four samples was assessed by utilizing *A. cepa* root tip meristems. In the control group, a gradual increase in the number of roots and root length was noticed. The average root length and number of roots in the negative control group at 24, 48, 72, and 96 hrs were 3.2 cm (*n* = 20), 4.2 cm (*n* = 30), 4.3 cm (*n* = 38), and 4.87 cm (*n* = 43), respectively (Table 4). All the formulations produced dose- and time-dependent growth suppression from 24 hrs. Incubation of bulbs in various concentrations of the extracts and the standard drug produced a growth hindering effect that was related to a decrease in the number of roots. Their cytotoxic effect was also noticeable in the form of decaying and shortening of roots both in extracts compared with standard drug cyclophosphamide. The Basam showed a significant decrease in root length as well as effective root decay property at 48, 72, and 96 hrs compared to 0 hr (*p* < 0.001). The root length and number of roots at 10 mg/ml of Basam were 2.77 (*n* = 12), 2.53 (*n* = 11), 2.20 (*n* = 10), 2.17 (*n* = 8), and 2 (*n* = 7) at 0, 24, 48, 72, and 96 hrs, respectively.

### 3.4. Antibacterial Activity

**Figure 2:**
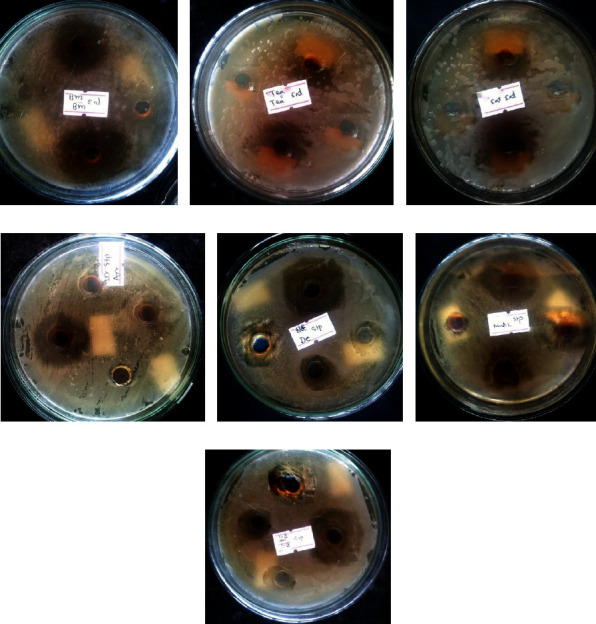
Zone of inhibition produced by the ethanolic extract of the samples (Basam, Raab Ga Yangzin Tea, and Sugmel-10) against *E. faecalis* and samples (Aru-18, Dadue, Mutik-25, and Serdok-11) against *S. aureus*. (a) Basam, (b) Raab Ga Yangzin Tea, (c) Sugmel-10, (d) Aru-18, (e) Dadue, (f) Mutik-25, and (g) Serdok-11.

The antibacterial activities of all nine formulations were examined by using a well-diffusion method against *Staphylococcus aureus, Enterococci faecalis, Klebsiella pneumoniae,* and *Pseudomonas aeruginosa*. Among them, *S. aureus* and *E. faecalis* were found to be highly sensitive, and their significant growth inhibition was seen in Dashel and Serdok-11 extracts, showing zone of inhibition 30 mm and 35 mm, respectively, while no activity was observed in Aru-18, Yungwa-4, and Dadue against *K.pneumoniae* and *P. aeruginosa*, respectively. On the other hand, ofloxacin as a standard antibiotic showed a higher zone of inhibition against *S. aureus* (32 mm)*, E. faecalis* (43 mm)*, K. pneumoniae* (41 mm), and *P. aeruginosa* (37 mm). Similarly, the antibacterial activity of standard antibiotic cefpodoxime against *S. aureus, E. faecalis, K. pneumoniae,* and *P.aeruginosa* was 28 mm, 31 mm, 25 mm, and 26 mm, respectively (Table 5). The zone of inhibition produced by the ethanolic extract of the samples against two bacterial strains is shown in Figure 2.

Ethanolic extracts that showed maximum antibacterial activity were taken for Minimum Inhibitory Concentration (MIC) assay. The MIC values of these compounds were measured by the broth dilution technique against *S. aureus* and *E. faecalis*. The results showed a higher inhibitory effect for Basam against *E. faecalis* (MIC 20 mg/ml). A lower antibacterial activity against *S. aureus* (MIC 80 mg/ml) was seen for Dashel. From the Minimum Bactericidal Concentration (MBC) test, ethanolic extract of Basam showed the least MBC value of 100 mg/ml against *E. faecalis* (Table 6).

## 4. Discussion

The traditional TM is commonly practiced in Nepal from time immemorial. In this study, we conducted an ethnomedicinal survey on Tibetan formulations. Based on the higher percentage of respondents, nine formulations are being used for stomach disorders, diabetes, and migraine. Ethanolic extract of all nine formulations was prepared, and their *in vitro* antioxidant, cytotoxic, and antibacterial activities were performed. Though ethnomedicinal studies were previously performed in medicinal plants used in Tibetan medicinal formula, antioxidant, cytotoxic, and antibacterial studies have not been reported on any Tibetan formulations. Limited pharmacological activities have been studied on these selected formulations. All India Institute of Medical Sciences (AIIMS) and the Tibetan Medical Astro Institute (TMAI) in Dharamsala, India, have conducted together an extensive study of Tibetan medicines such as Aru-18 (18 component formula), Yungwa-4, Sugmel-19, and Kyura-6 on diabetic patients (type 2 diabetes) [39]. Diabetes is the most commonly observed chronic disease in Tibetan medical dispensaries [40]. In diabetes mellitus, Chinni-Aru-18, Kyuru-6, Sugmel-10, and Yungwa-4 were also used [41]. Individualized combinations of Tibetan formulations (Aru-18, Yungwa-4, Sugmel-19, and Kyuru-6) also have a glucose-lowering effect [42]. The first regimen of Tibetan medicine contained mainly of Dashel Dhuetsema, Dashel Sodhuen, Dangney, Dagshun Gupa, Kyuru-Drukpa, Yungwa Shithang, Aru Chupa, and Sangdak after blood cancer was diagnosed in Tibetan medical treatment in 2000. Also, in Tibetan medical diagnosis, Sangdak Dharyaken, Gawa Chudruk, Dashel Sodhuen, Gurgum Chusum, Yukar, and Khyunga and precious pills (Rinchen Tsodru Dashel and Rinchen Mangjor Chenmo) were prescribed in stomach cancer [43]. The oil-based Basam medicine preparation method in the work by Chakhar Geshe Lobsang Tsultim includes information on preparing medicines for the treatment of diseases by infections [44]. Mutik-25 has been practiced in the clinic for more than 1000 years. It is highly appreciated by patients and proved to have significant effects on neurological disorders [30]. Dadue is suggested to boost immunity power against some viral infections such as COVID-19 [45].

*Terminalia chebula* Retz. (Aru ra), *Terminalia bellirica* (Gaertn.) Roxb. (Baru ra), and *Phyllanthus emblica* L. (Kyuru ra) are commonly used ingredients in Tibetan medicines commonly known in Tibetan as Aru-Baru-Kyuru similar to triphala in Ayurveda [46]. The decoction of these three fruits is used in contagious diseases and has blood cleansing properties, and powdered compounds are used in constipation, abdominal bloating, and digestive disorders [47]. Flavones have been used to prevent hypoglycemia, scavenge free radicals, inhibit the growth of tumors, and assist in bacteriostasis in traditional TM. Numerous traditional TMs, despite, have unknown active ingredients [3]. *Myristica fragrans* Houtt. is used against poor appetite and heat loss in the stomach and maintains mental stability. *Phyllanthus emblica* L. acts as a diuretic and is used in the treatment of polyuria. *Swertia petiolata* D. Don is used against inflammation of the stomach and kidneys. Metals and detoxified mercury are usually used in Tibetan medicine [48]. Other essential constituents of the medicine are minerals and gems which constitute 5 to 7% of medicinal substances [29]. *T. chebula* Retz., *T. bellirica* (Gaertn.) Roxb. (Baru ra), *P. emblica* L., *M. fragrans* Houtt., *P. emblica* L., *S. petiolata* D. Don, animals and their products, metals, etc. are also commonly used ingredients in our selected formulations, as seen in Table 1.

Brag-zun (mineral pitch in English and shilajit in Sanskrit) is used in numerous traditional medicines for treating various disorders such as stomach ulcers, dysentry, liver diseases, and parasitic diseases, and the Bhutanese variety Brag-zun showed antioxidant and antimicrobial properties. Brag-zun is used in preparing Bhutanese traditional medicine in combination with Basam, which is used in kidney diseases [49]. In previous studies, Shilajit was also found to have antidiabetic, anti-inflammatory, and antiulcerogenic effects [50–52]. As shown in Table 1, the mineral pitch is also an ingredient in some of our selected samples such as Dadue, Dashel, and Serdok-11, and our ethnomedicinal survey justified that these were the most used three formulations in stomach disorders and migraine. Pearl is also used in various precious medicines in the old traditional Tibetan system of medicine called Sowa Rigpa, such as Mutik-6, Mutik-25, and Mutik-70 [53].

Free radicals are involved in numerous disorders such as neurodegenerative diseases, cancer, and HIV-AIDS. The scavenging power of antioxidants is helpful for the management of these diseases. DPPH free-radical method is an easy, sensitive, and rapid way to examine the antioxidant activity of a particular compound or extracts [54]. Some commonly used traditional preparations have inbuilt antioxidant activity, and their therapeutic potential can be partially attributable to their antioxidant activity. Traditional TM depends greatly on plant-derived compounds and extracts where plant materials are rich in antioxidants such as flavonoids, polyphenols, and vitamin C [55]. So, we determined the antioxidant activity of such common formulations. All nine formulations showed good antioxidant activity. Among them, Sugmel-10 has the highest DPPH free-radical-scavenging activity as compared to ascorbic acid.

**Figure 3:**
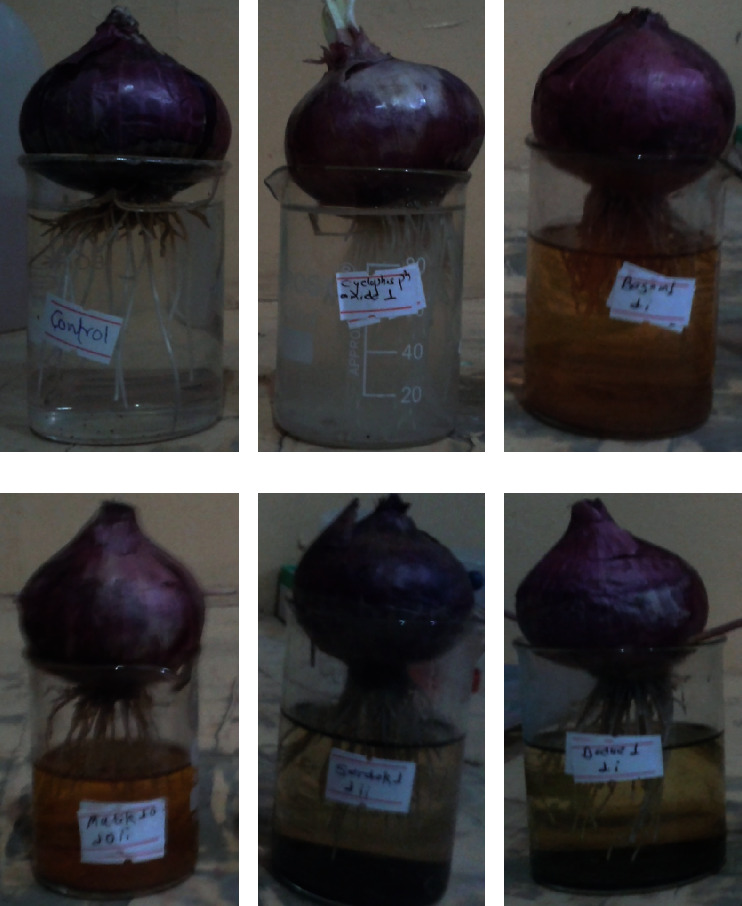
Effect of different ethanolic extracted samples on the root length and number in *Allium cepa* bulbs after 96 hrs of incubation. (a) Control, (b) cyclophosphamide, (c) Basam, (d) Mutik-25, (e) Serdok-11, (f) Dadue.

There is very little documentation regarding the efficacy of TM for treating cancer. Mineral and herbal formulas are prominently used in curing cancer patients [43]. Some promising effects of TM for cancer therapy imply that it might be a good source of chemotherapeutic agents against cancer. Hence, we evaluated the cytotoxic potential of four Tibetan formulations for the first time by using the *A. cepa* root tip meristem model. The cyclophosphamide, a mitotic inhibitor, was used as a positive control where all four formulations showed significant cytotoxic activity. Thus, we assumed that our bioactive extracts might have exhibited cytotoxic activity through mitotic inhibition in onion root meristems (Figure 3). Further comprehensive studies are necessary to determine the precise mechanism of action.

To our knowledge, the antimicrobial activity of traditional TM used in Nepal has not been performed. Data showed that these formulations are prepared from different plant extracts having antibacterial properties. Thus, to justify their antibacterial property, we performed antibacterial assay through well-diffusion and broth dilution methods against *S. aureus* and *E. faecalis*. From this study, the result of MIC and MBC confirmed the antibacterial activity of Basam, Dadue, and Mutik-25. In addition, further study is needed to investigate their potency, efficacy, and mechanism of action to develop new antibiotics from these TM.

The clinical research of traditional TM in western industrialized countries is scarce but shows interesting results. Also, larger trials are needed by applying a better research methodology [56]. The crucial issue in the modernization of TM involved disclosing the active ingredients along with working mechanisms of Tibetan materia medica (TMM) and preparations in treating various diseases. So, higher importance has been linked to scientific research on those TMM and its preparations [57].

The limitations of this study include the reluctance of respondents towards the queries, use of multiple formulations owing to the comorbidities of the respondents, and the unwillingness of Amchis to share detailed information regarding the uses of traditional Tibetan formulations. Moreover, the use of multiple ingredients and lack of standardization of the formulations could compromise the quality and effectiveness of these Tibetan medicines. This study carried out for the first time, thus, reveals the need for a more ethnomedicinal study, proper documentation of the traditional Tibetan medicine practices, and to conduct quality control of the formulations.

## 5. Conclusions

In this study, assessments of the reported ethnomedicinal survey showed that formulations (Aru-18, Basam, Dadue, Dashel, Mutik-25, Raab Ga Yangzin Tea, Serdok-11, Sugmel-10, and Yungwa-4) were used for the treatment of stomach disorders, diabetes, and migraine. From the free-radical-scavenging test, the most remarkable antioxidant potential was seen in Sugmel-10. Among them, Basam, Dadue, Mutik-25, and Serdok-11 exhibited significant dose- and time-dependent cytotoxic activity in the *A. cepa* root meristem model. Also, from the antibacterial test, Basam demonstrated the highest inhibitory effect and the least MBC value against *E. faecalis.* Thus, these results encourage additional biological studies *in vitro* and *in vivo* with different cell lines to evaluate the possibilities of using the Tibetan formulations for the development of novel and revolutionary drugs of pharmacological interest.

## Figures and Tables

**Table 1:** Uses and applications of nine selected Tibetan formulations.

S. no.	Formulations	Ingredients	Uses/actions	Applications	References
1	Aru-18 (myrobalan 18)	*Carthamus tinctorius, Caesalpinia bonducella, Cupressus torulosa, Elettaria cardamomum, Eugenia jambolana, Malva verticillata, Mucuna prurita, Rubia cordifolia, Swertia chirata, Symplocos crataegoides, Terminalia chebula, Verbascum thapsus,* vermilion, and crabshell	Inflammation of the kidney; kidney disorder giving rise to stooping; pain in hip and waist region; imbalance of kidney channels	2-3 g once daily in the afternoon with warm water	[29]

2	Basam (medicinal butter)	*Angelica species, Asparagus spinosissimus, Emblica officinalis, Mirabilis himalaica, Polygonatum cirrhifolium, Terminalia belerica, Terminalia chebula, Tribulus terrestris,* clarified butter, honey, and milk	Kidney diseases; leg cramps; tonify body, promotes longevity; pain in the bones, hip joint, and lower back muscles; male-specific diseases such as impotence, prostatitis, and decreased libido	1–4 g daily with warm water	[29, 30]

3	Dadue	*Carthamus tinctorius, Crocus sativas, Dracocephalus tanguiticum, Inula racemosa, Saussurea lappa,* calcium carbonate, iron powder, and mineral pitch	Liver disorder, gastric problem, food poisoning, indigestion, chronic fever, colic pain, eye problem, all kinds of chronic diseases, and general tonic	1-2 pills daily in the morning or evening with warm water	[30]

4	Dashel	*Aconitum heterophyllum, Aconitum orochryseum, Adhatoda vasica, Amomum subulatum, Bainbusa textilis, Beaumontia grandiflora, Carthamus tinctorius, Commiphora mukul, Corydalis stracheyi, Crocus sativus, Cynanchum thesioides, Dracocephalus tanguiticum, Elettaria cardamonium, Emblica officinalis, Eugenia caryophyllata, Cerinthe gymnandra, Herpetospermum pedunculosum, Inula racemosa, Lagotis kunawurensis, Meconopsis species, Moschus moschiferus, Myristica fragrans, Pedicularis oliveriana, Picrorhiza kurroa, Piper longum, Pterocarpus santalinus, Punica granatum, Rheum spiciforme, Santalum album, Saussurea costus, Saussurea lappa, Saxifi aga umhellulata, Strychnos nux-vomica, Syzigium aromaticum, Taraxacum officinale, Terminalia chebula, Ursus thibetanus, Vincetoxicum sibiricum,* calcitum, mineral pitch, and smithsonite (calamine)	Gastrointestinal diseases; gastrointestinal tumor; cholecystitis; hepatitis; nausea; belching; constipation; chronic diseases of the stomach, liver, and skin; anti-inflammatory action in the gastrointestinal tract	1-2 g in the morning or evening with warm water	[30]

5	Mutik-25 (Pearl 25)	*Amomum subulatum, Bambusa textilis, Bos taurus domesticus, Carthamus tinctorius, Cinnamomum zeylanicum, Cuminum cyminum, Emblica officinalis, Elettaria cardamomum, Eugenia caryophyllata, Gmelina arborea, Margaritum, Myristica fragrans, Nigella sativa, Piper longum, Polygonum aviculare, Potamom yunnanensi, Pterocarpus santalinus, Punica granatum, Saussurea lappa, Terminalia belerica, Terminalia chebula, Malva verticillata, Moschus moschiferus, Santalum album,* and vermiculite	Hypertension, stroke neuralgia, hemiplegia, palpitation, tranquilizer, facial paralysis, unconsciousness, delirious mania	1-2 pills one hour before or after meals with warm water daily	[30]

6	Raab Ga Yangzin Tea (herbal tea)	*Carthamus tinctorius, Elettaria, Rosa bronunii, Rubus hoffmeiteriannus, Symplocos paniculata, and Zingiber officinalis*	Provides body energy; promotes skin health; appetite; relaxation; assists in cold, flu, and poor digestion	Pour hot water on the teabag, infuse for few minutes, then relish it purely or with salt or sugar	[31]

7	Serdok-11	*Carthamus tinctorius, Crocus sativa, Cuminum cyminum, Emblica officinalis, Herpetospermum caudgerum, Myristica fragrans, Punica granatum, Rosa bronunii, Saussurea lappa, Terminalia chebula,* and mineral pitch	Acute and chronic diseases of the liver and gall bladder (cirrhosis, cholecystitis, and gallstone disease); protects liver cells; restores the function of the pancreas; normalizes metabolism	2 pills daily with hot water	[30, 32]

8	Sugmel-10 (cardamom 10)	*Caesalpinia bonducella, Elettaria cardamomum, Eugenia jambolana*, *Hedychium spicatum, Malva verticillata, Piper longum,* crab shell, and sodium chloride	Kidney disorders, removes kidney stones, clears obstruction of the urinary tract, removes tumors and stones from the urinary bladder	2-3 g daily at night with hot or warm water	[30]

9	Yungwa-4 (decoctions of turmeric)	*Berberis dictyophylla, Curcuma longa, Emblica officinalis, and Tribulus terrestris*	Antipyretic, diuretic, inflammation of the urethra	3–5 g decocted to one-third water level and taken twice daily	[29]

**Table 2:** Dosage form and percentage of respondents for stomach disorders, diabetes, and migraine of nine major Tibetan formulations.

S. no.	Formulations	Dosage form	% of respondents		
			Stomach disorders	Diabetes	Migraine
1	Aru-18	Pills	—	11.25	8.75
2	Basam	Pills	8.75	—	—
3	Dadue	Pills	7.5	—	—
4	Dashel	Pills	12.5	—	—
5	Mutik-25	Pills	—	—	6.25
6	Raab Ga Yangzin Tea	Tea pouch	7.5	—	—
7	Serdok-11	Pills	—	—	10
8	Sugmel-10	Pills	—	8.75	—
9	Yungwa-4	Pills	—	5	—

**Table 3:** DPPH free-radical-scavenging activity of the samples.

% Scavenging activity of samples					
Sample/concentration	0.1 *μ*g/ml	1 *μ*g/ml	10 *μ*g/ml	100 *μ*g/ml	**IC**_**50**_ *** μ*g/ml**
Aru-18	5.79 ± 0.84	16.9 ± 0.45	93.79 ± 0.27	95.40 ± 0.20	4.98
Basam	8.48 ± 1.88	21.32 ± 9.06	92.41 ± 0.27	95.45 ± 0.37	4.8
Dadue	3.46 ± 0.62	22.81 ± 0.91	91.99 ± 0.62	93.72 ± 2.18	5.15
Dashel	5.55 ± 0.64	33.33 ± 2.95	87.09 ± 0.35	93.49 ± 0.45	4.95
Mutik-25	15.17 ± 4.51	27.71 ± 11.35	92.59 ± 0.37	92.53 ± 0.45	4.33
Raab Ga Yangzin Tea	21.74 ± 2.73	29.62 ± 0.45	90.62 ± 0.45	92.47 ± 0.17	4.09
Serdok-11	7.10 ± 1.93	20.37 ± 2.51	92.11 ± 0.17	94.98 ± 0.71	4.91
Sugmel-10	38.89 ± 3.12	48.74 ± 1.59	91.09 ± 0.62	95.34 ± 0.47	1.8
Yungwa-4	3.52 ± 1.36	21.62 ± 1.27	87.51 ± 0.62	93.19 ± 0.17	5.25
Ascorbic acid	3.46 ± 1.21	16.12 ± 1.72	96.41 ± 0.53	96.96 ± 0.31	4.93

Data are expressed as mean value ± standard deviation (*n* = 3).

**Table 4:** Root length and number of *Allium cepa* obtained after incubation with ethanolic extracts of the samples and standard drug.

Groups	Concentration	Root length (cm)				
		0 (hr)	24 (hrs)	48 (hrs)	72 (hrs)	96 (hrs)
Control	Distilled water	3 ± 0.29 (*n* = 10)	3.20 ± 0.15 (*n* = 20)	4.20 ± 0.12 (*n* = 30)	4.30 ± 0.06 (*n* = 38)	4.87 ± 0.07 (*n* = 43)

Basam	1 mg/ml	2.47 ± 0.29 (*n* = 13)	2.23 ± 0.15 (*n* = 14)	2.07 ± 0.18^*∗∗∗*^ (*n* = 17)	1.70 ± 0.15^*∗∗∗*^ (*n* = 18)	1.70 ± 0.15^*∗∗∗*^ (*n* = 22)
	10 mg/ml	2.77 ± 0.15 (*n* = 12)	2.53 ± 0.09 (*n* = 11)	2.20 ± 0.12^*∗∗∗*^ (*n* = 10)	2.17 ± 0.09^*∗∗∗*^ (*n* = 8)	2 ± 0.12^*∗∗∗*^ (*n* = 7)

Dadue	1 mg/ml	2.67 ± 0.09 (*n* = 13)	2.67 ± 0.09 (*n* = 14)	2.57 ± 0.07^*∗∗*^ (*n* = 16)	2.43 ± 0.09^*∗∗∗*^ (*n* = 18)	2.33 ± 0.09^*∗∗∗*^ (*n* = 19)
	10 mg/ml	3 ± 0.29 (*n* = 14)	3 ± 0.29 (*n* = 13)	2.90 ± 0.23^*∗∗*^ (*n* = 12)	2.70 ± 0.21^*∗∗*^ (*n* = 10)	2.60 ± 0.15^*∗∗∗*^ (*n* = 9)

Mutik-25	1 mg/ml	2.90 ± 0.49 (*n* = 14)	2.73 ± 0.43 (*n* = 16)	2.73 ± 0.43^*∗∗*^ (*n* = 17)	2.30 ± 0.44^*∗∗∗*^ (*n* = 20)	2.27 ± 0.43^*∗∗∗*^ (*n* = 24)
	10 mg/ml	2.90 ± 0.35 (*n* = 14)	2.93 ± 0.34 (*n* = 14)	2.57 ± 0.35^*∗∗*^ (*n* = 12)	2.50 ± 0.29^*∗∗∗*^ (*n* = 10)	2.33 ± 0.17^*∗∗∗*^ (*n* = 10)

Serdok-11	1 mg/ml	4.10 ± 0.15 (*n* = 13)	4.73 ± 0.30^*∗∗∗*^ (*n* = 14)	4.57 ± 0.15 (*n* = 16)	4.47 ± 0.20 (*n* = 18)	4.37 ± 0.19 (*n* = 19)
	10 mg/ml	2.63 ± 0.09 (*n* = 11)	2.57 ± 0.13 (*n* = 10)	2.53 ± 0.17^*∗∗∗*^ (*n* = 9)	2.47 ± 0.13^*∗∗∗*^ (*n* = 8)	2.37 ± 0.13^*∗∗∗*^ (*n* = 7)

Cyclophosphamide	1 mg/ml	2.97 ± 0.03 (*n* = 12)	2.97 ± 0.09 (*n* = 14)	2.90 ± 0.12^*∗∗*^ (*n* = 14)	2.53 ± 0.18^*∗∗∗*^ (*n* = 16)	2.40 ± 0.23^*∗∗∗*^ (*n* = 18)
	10 mg/ml	3.57 ± 0.43 (*n* = 13)	3.30 ± 0.44 (*n* = 11)	3.10 ± 0.44^*∗*^ (*n* = 10)	2.87 ± 0.43^*∗∗*^ (*n* = 8)	2.67 ± 0.55^*∗∗∗*^ (*n* = 6)

Notes: all experiments were performed with three replicates, and data were expressed as mean ± standard error of the mean. Statistical significance is given for comparison of root length obtained at 24, 48, 72, and 96 hrs with respect to the control (distilled water) ^*∗*^*p* < 0.05, ^*∗∗*^*p* < 0.01, ^*∗∗∗*^*p* < 0.001; *n*: root number.

**Table 5:** Inhibition zone of the ethanolic extract of the samples against four bacterial strains at a concentration of 100 mg/ml.

Samples	Microorganisms			
	ZOI (mm)			
	*S. aureus*	*E. faecalis*	*K. pneumoniae*	*P. aeruginosa*
Aru-18	13	14	—	—
Basam	18	33	—	15
Dadue	15	24	—	—
Dashel	30	25	—	15
Mutik-25	21	12	—	16
Raab Ga Yangzin Tea	15	25	—	20
Serdok-11	33	35	—	18
Sugmel-10	14	—	13	13
Yungwa-4	14	27	—	—
Cefpodoxime^a^	28	31	25	26
Ofloxacin^a^	32	43	41	37

^a^Used as standard antibiotics; data represent the zone of inhibition (ZOI) in mm.

**Table 6:** MIC and MBC values of ethanolic extracts of the samples against two different bacterial strains.

Microorganism	Samples	MIC (mg/ml)	MBC (mg/ml)
*E. faecalis*	Basam	20	100
	Dadue	50	120

*S. aureus*	Dashel	80	>150
	Mutik-25	50	120
	Serdok-11	50	>150

## Data Availability

The data used during the study will be available from the corresponding author upon request.
